# Access to patient oriented information—a baseline Endo-ERN survey among patients with rare endocrine disorders

**DOI:** 10.1007/s12020-021-02654-9

**Published:** 2021-02-18

**Authors:** Violeta Iotova, Camilla Schalin-Jäntti, Petra Bruegmann, Manuela Broesamle, Johan De Graaf, Natasa Bratina, Vallo Tillmann, Alberto M. Pereira, Olaf Hiort

**Affiliations:** 1 Endo-ERN Work Package ‘Education & Training’ Paediatric Chair; Deptartment of Pediatrics, Medical University of Varna, 55“M. Drinov” Str, 9002 Varna, Bulgaria; 2 Endo-ERN Work Package ‘Education & Training’ Adult Chair; Endocrinology, Abdominal Center, University of Helsinki and Helsinki University Hospital, Helsinki, Finland; 3 Endo-ERN Work Package ‘Education & Training’ European Patient Advocacy Group (ePAG) representative co-chair, Endo-ERN, Leiden, The Netherlands; 4 Department of Endocrinology, Diabetes and Metabolic disorders, University Medical Center, University Childrens Hospital, Ljubljana, Bohoriceva 20, 1000 Ljubljana, Slovenia; 5 Children’s Clinic, Tartu University Hospital, Tartu, Estonia; 6 Adult Chair and coordinator of Endo-ERN, Division of Endocrinology, Department of Medicine, Leiden University Medical Center, Leiden, the Netherlands; 7 Paediatric Chair and deputy coordinator of Endo-ERN, Division of Paediatric Endocrinology and Diabetes, Department of Paediatric and Adolescent Medicine, University of Lübeck, Lübeck, Germany

**Keywords:** Education, Patients, Parents/caregivers, European patient advocacy group, Rare endocrine disease, Endo-ERN

## Abstract

**Aim:**

To perform a baseline survey on condition-specific information access among patients/parents/caregivers with rare endocrine disorders (RD) in Europe.

**Methods:**

Electronic invitation to participate in a survey (19 questions) was sent to 120 patient advocacy groups (PAGs), and further distributed to 32 European countries.

**Results:**

A total of 1138 respondents from 22 countries (74% women), aged between 1 year (parents) and 70 years, participated. The Netherlands, France, Germany, Italy and France had highest participation rates. All Main Thematic Groups (MTGs) were represented; the adrenal (32%), pituitary (26%) and thyroid (22%) were the most common. The majority of the respondents got information from their endocrinologist (75%), PAGs (37%) and expert reference centre (22%); 95% received information in their mother tongue. Leaflets (70%), infographics (65%), webinars (60%) and Internet films (55%) were preferred ways of learning. Respondents relied mostly on materials by PAGs and alliances (79%), rather than from specific international RD sites (15%). Fifty-six percent used Facebook, and 37% other social media, with a significant age difference (<40/>40 years) among non-users, 19% vs. 36%, *p* < 0.0001. Of all, 685 answered questions on informational materials for children−79% wanted materials that can be used by the children themselves. There was significant age difference (<40 years/>40 years) in the willingness to help create new educational materials; 49% vs. 34%, *p* < 0.001.

**Conclusions:**

Our current patient information access survey provides a sound basis for further planning and execution of educational and teaching activities by Endo-ERN.

## Background

European Reference Networks (ERNs) for rare disorders (RD) are based on the directive 2011/24/EU of the European Parliament governing the right of European citizens to have access to cross-border healthcare (https://eur-lex.europa.eu/LexUriServ/LexUriServ.do?uri=OJ:L:2011:088:0045:0065:en:PDF). ERNs are virtual networks linking expert reference centres across Europe. Operation started in 2017 with the main goal to exchange knowledge, resources and competence to tackle the rare conditions paradigm, and secure rapid improvement of RD care. At the same time, ERNs are a 5 year pilot project to test the above hypothesized goal in real life conditions that are still so diverse in the European Union and even more diverse globally. The process was inspired by the patients in the early 1980s. A complex development of care and policies at national level started followed by organizational and hierarchical measures at the European level, and lead to the idea of creating virtual reference networks [for review, see [1]]. Participatory approach to patients is a must from the very beginning. At the very heart of the idea is the strive of patients and their collective European patient advocacy group representatives (ePAGs) to decrease time to diagnosis, to improve treatment and follow-up, and to increase support of RD patients working together with the medical community [2–4]. One of the continuous efforts of the ePAGs is to measure patients and their representatives (parents/caregivers/stakeholders) opinion (https://www.eurordis.org/content/current-surveys).

Timely and quality information and education is important for patient empowerment [5], and coping with the sense of social isolation receiving a rare diagnosis is crucial for better outcome [6]. PAG representatives promote knowledge generation (research and education) most successfully through partnership models [7] thus facilitating acceleration of quality improvement of RD healthcare. From the beginning, every ERN was free to decide how to organize and secure their participation.

The European Reference Network on Rare Endocrine Conditions (Endo-ERN) unites 86 health care providers (HCPs) from 27 European countries, currently including the UK. The core principles are *equality* between paediatric and adult patients, between paediatric and adult health care professionals, and equal representation (adult and paediatric) of ePAGs. Endo-ERN promotes diversity and strives to achieve cross-borders health equality and better care for patients with endocrine rare conditions throughout life-span.

The organizational structure is based on eight main thematic groups (MTGs), namely: MTG1 Adrenal; MTG2 Disorders of Calcium and Phosphate Homeostasis; MTG3 Genetic Disorders of Glucose and Insulin Homeostasis; MTG4 Genetic Endocrine Tumor Syndromes; MTG5 Growth and Genetic Obesity Syndromes; MTG6 Pituitary, MTG7 Sex Development and Maturation, and MTG8 Thyroid. Every MTG strived to have four chairs—a paediatric, and an adult professional, and two ePAG representatives, favorably a paediatric and an adult one. The structure is complemented with five “horizontal” Work Packages (WPs). Work package 1 (WP1) Education & Training has the same type of governance as described above consisting of four chairs, and major tasks are to collect, qualitatively assess, align and boost education and teaching in the field of rare endocrine diseases.

Education and training in the field of rare endocrine diseases is aiming at health care professionals, patients, stakeholders, and society. Apparently, it could have different dimensions and focus based on the audience [8]. In order to plan further actions how to generate knowledge within and beyond the Network, the first step was to map the situation at baseline. Health professionals’ relevant education and training resources were evaluated (Iotova et al., submitted), and for patients, the most relevant current informational sources were evaluated with view to use these for further knowledge generation and dissemination.

The aim of this study was to assess which informational sources patients/parents/caregivers with rare endocrine conditions in Europe preferred at the time of initiation of Endo-ERN.

## Materials and methods

A survey was prepared (EUSurvey, https://ec.europa.eu/eusurvey/home/about) consisting of 12 main questions, some of which had subsections (overall 19 questions). It was entirely constructed by the WP1 ePAG Co-chairs (M.B. and P.B.), who are very experienced in the patient advocacy field [9].

### Data collection and analysis

The survey began distribution on 09/03/2018 and the last response was received on 22/05/2018.

An invitation e-mail was sent to 120 patient advocacy groups (PAGs) in 20 European countries, and then re-distributed via Facebook, Twitter, and Messenger to cover a total of 32 countries. To facilitate participation, the survey was offered for translation to patients/advocacy groups in all European countries, and was thereafter translated in seven European languages (Bulgarian, Dutch, English, French, German, Italian, Spanish), with the voluntary work of patients and with the help of local HCP Network representatives. At present, these documents and links to the individual patient groups are collected on the CIRCABC Platform of the EU, sorted by country as well as by affiliation to the individual MTGs. The survey explored the use of informational resources among endocrine RD patients/parents/caregivers and relevant stakeholders in order to enable the Network to develop these further in the next 3 years of its development.

Informed consent was actively sought by every participant by asking them whether they are happy to continue the survey after initial explanation about survey’s aim and scope. By ticking the “yesˮ box all participants agreed to participation and to continue the survey.

Analysis was done automatically with the DIGIT-EUROSURVEY system. Statistical significance was assessed by Chi-square test. Level of significance was set at *p* < 0.05.

## Results

### Characteristics of the study cohort

#### Participating countries

A total of 1138 respondents participated, the majority from five countries whose ePAGs had representatives at the Network—The Netherlands, France, Germany, Italy and UK—987 (16% of all addressed countries, 87% of all answers) (see supplementary Table 1). Fewer than five responses came from 28% of the participating countries. No answers were received from patients from Bosnia and Herzegovina, Croatia, Cyprus, Czech Republic, Estonia, Latvia, Lithuania, Poland, Macedonia and Slovak Republic (31% of all surveyed countries).

#### Gender, age distribution, and participation according to MTG affiliation

**Fig. 1:**
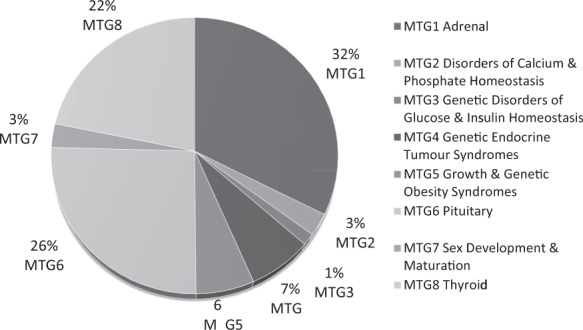
Distribution of patients according to their MTG affiliation within the Endo-ERN

(Figure 1) Respondents answered in their role as a patient or a parent/caregiver on behalf of the patient. Most participants were aged between 41 and 70 years (63%), and 74% were women. Two-thirds of the participants were affiliated to 3 of the 8 Endo-ERN MTGs.

### Sources of information

#### Language

According to 95% of the respondents, patients primarily seek and receive information in their native language.

#### Primary source of information

**Fig. 2:**
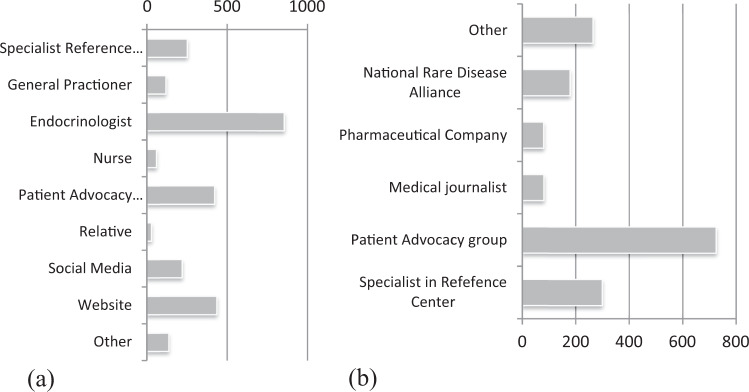
Answers to the question “Where do you get the information about the rare disease?” (**a**) and “Who published the information?” (**b**) (more than one answer was possible)

(Figure 2a) Most participants received information from their endocrinologist (75%), followed by PAGs (37%) and expert reference centres (22%).

#### Preferred ways of learning about rare endocrine disorders

On a Likert scale, most respondents considered leaflets as very helpful (70%), followed by infographics (65%), webinars (60%) and Internet films (55%).

#### Current access to information

Regarding currently used sources, leaflets (34%) and “other” (unspecified) sources were equally common (34%), followed by Internet movies (22%). Although infographics were ranked as a highly preferred source (78%), only 13% currently use infographics, while 18% participated in webinars. There was no statistically significant difference in the use of any of these sources by age.

#### Reliability

**Fig. 3:**
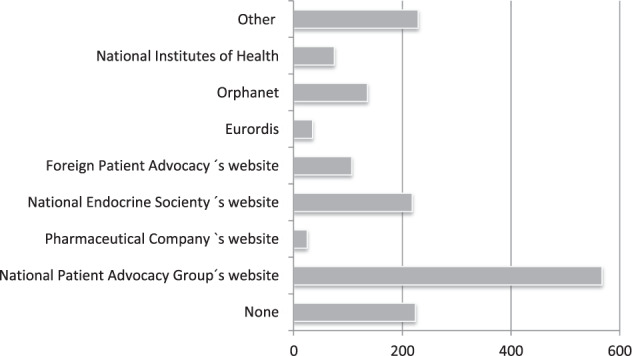
The distribution of the Internet websites currently used by patients with rare endocrine diseases

Patients/respondents confined predominantly in materials published by ePAGs and alliances (79%). The relative share of the materials provided by reference centres was 26%, while very few mentioned medical journalists (7%) or pharmaceutical companies (7%) as their informational source (Fig. 2b). These findings are further supported by the data on currently used websites (Fig. 3). The majority, 49%, used websites of the national PAGs, followed by various official medical websites. Specifically, designed patient sites for RD such as Eurordis, Orphanet, and others were not among preferred choices, in total 22%. Currently, 20% of patients/parents/caregivers did not use any site, while 44% acquire information as a one-way off activity, without any further updates.

#### Social media

Half of the respondents, 56%, were active on Facebook, almost 17% used Whatsapp, and the others used Twitter, Instagram or other forms of social media (SM). In contrast, 30% did not use any of the SM channels listed in the survey (several answers were possible).

**Fig. 4:**
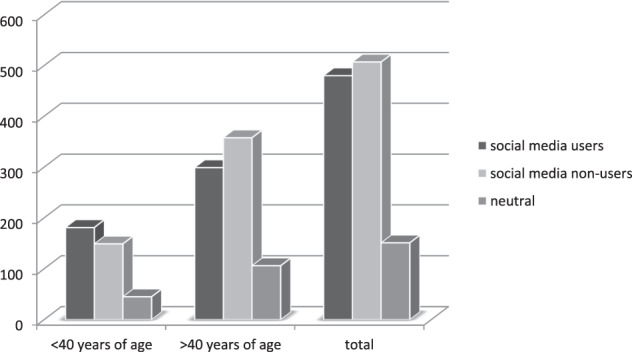
Distribution of social media users among patients within Endo-ERN by age

When asked whether they would impart general information about their disorder via SM, 44% considered this very unlikely/unlikely, while 42% responded with “very likely/likely”. Further analysis by age showed that participants <40 years of age (*n* = 375) were more likely to use SM than those >40 years of age (*n* = 763) (Fig. 4). This result was further supported by the significantly higher age of complete non-users of SM, 19% of respondents <40 years, vs. 36% >40 years (*χ*^2^ = 35.07, *p* < 0.0001).

### Educational materials for children

A total of 685/1138 participants responded to survey questions regarding the need for informational materials for children. The vast majority (79%) expressed their wish for special materials that can be used by the children themselves.

### Satisfaction with available information and willingness to help create new materials

On a Likert scale, in the whole group 57% were very satisfied/somewhat satisfied with the available information, while 28% were very dissatisfied/dissatisfied. The analysis by age of the participants (</>40 years) showed that those <40 years were much more dissatisfied with available informations (*χ*^2^ = 9.19, *p* = 0.002).

When further asked whether they would participate in creating new materials, 61% responded that they would not want to be engaged in such activities. However, when stratified by age, 49% of people <40 years would engage in creating new informational materials vs. 34% of people >40 years (*χ*^2^ = 22.89, *p* < 0.0001).

## Discussion

Today, patient education is recognized as an essential tool for improving care of patients with RD [10]. Recently, Pelentsov et al. [11] defined educational needs of parents of children with RD (62%) as the second priority after social needs (72%). Our baseline Endo-ERN information access patient survey that included 1138 participants is, to our knowledge, the largest attempt so far to capture patient educational access data in Europe. This paper provides evidence that information access has developed lately to a large extent, especially as a powerful lever of patient empowerment [12, 13]. This is probably a result of the concerted action in the recent decades to unite RD patients and create a shared platform for common goals.

The development of the Internet is acknowledged as a major contributor to these goals [14]. In practice, it currently reaches all communities and assures better access to information [6, 15]. The level of inclusion of patients within structures called on stage to secure addressing needs and raising the patients’ voice whenever their health care, needs and rights are concerned is very diverse. A third of the countries in Europe, the majority of which are European Union member states, seem not to be active and not a single patient voice responded from these countries. Furthermore, fewer than five answers were received from another 45% of countries. Thus, this study is not representative for the whole of Europe but rather represents the more organized and professionally advanced parts of it, and for ePAGs that already have reached a certain level of maturity. The same pattern is seen among participants in other RD surveys (https://www.eurordis.org/voices. *Accessed Oct 20th, 2020*). This shows that Endo-ERN needs to invest more effort and planned action to reach out to the still silent patient communities in these countries.

It was of no surprise that most of the survey respondents were female. This has been replicated many times in different previous studies in rare patient communities [6, 15, 16]. The study captured primarily a mature age cohort of both patients and parents/caregivers of patients with RD. Since new informational technologies have emerged at a fast rate in the last decade, and because we aimed primarily to collect information to plan future action, further analysis based on age was required.

As a uniform finding, patients/parents/caregivers obtain information about their disease from their primary doctor—the endocrinologist. Importantly, the respondents rank ePAGs second and expert reference centres third. This underlines the important role of ePAGs, specifically of their professional development and maturation as lay-men-experts, since their first emergence more than 25 years ago [1] and the quality of information they provide [17]. At the very beginning of the Endo-ERN this finding also importantly shaped Network outreach and efforts to bolster knowledge distribution.

A unanimous result, that was independent of age, was the current preference of leaflets as a primary educational resource, as well as the providing doctor/expert centre as the informational source, followed by Internet films and webinars. We assume that the latter is connected to the many useful and well-designed materials made available through the activity of health care professionals and the ePAGs that are already reaching patients (Endo-ERN current collection at https://endo-ern.eu/patients/patient-information-materials/) [18]. A prerequisite of success is the availability in the native language, still a major preference among patients according to respondents of the current survey (95%).

The result on confidence in information provided by the PAGs are also very important. It not only tells how well developed they are—at least in some parts of Europe—but also shows that one of the primary goals of the RD movement—overcoming social isolation and seeking new ways for better treatment and care, is at least partially fulfilled. It is evident that the distribution of education and know-how at the national level, and in local languages as well as actively attracting leadership requires further top-down action [7]. Other challenges to tackle are the passive attitude of a significant number of patients/parents/caregivers to the RD movement, and the paucity of continuous and sustainable education [19].

The results of the assessment of attitudes to and use of SM in the current survey are intriguing. The preference for exchanging general information about the disorder via SM, on the one hand, and the aversion to SM on the other hand, were equally common in our study cohort. However, the majority of those who would not share information through SM were patients/parents/caregivers >40 years of age (*χ*^2^ = 35.07, *p* < 0.0001). Younger respondents use SM significantly more, but are at the same time more dissatisfied with the available information in general. This finding, as well as the predominance of female respondents, is in line with those of other studies. DeHoff et al. [6] found that nearly all women aged 18–29 in the USA used SM, and find social support there [20]. Social media are expected to become more and more useful for diagnosing rare disorder patients [21].

In the future, to achieve faster patient outreach, especially to younger ones, more educational content of sufficient quality could be distributed through SM to serve not only as an educational and social support resource but also for sharing best practices [10]. SM could also serve as means of studying RD per se [2, 19], following examples from other more common disorders [22]. The major source should be the PAGs’ tools in the local languages since patients mostly need peer support with easy access to reliable information that is meaningful to them [11]. An important goal is the development of a collection of specific education and training materials from the MTGs. Of note, younger patients/parents/caregivers are not only in need of, but also are more inclined to engage in creating new materials. Further attention has to be invested in patients who currently do not use any informational source in order to create ways of reaching out and helping them as well. Social media seems to hold great potential as it is a combination of emotional and informational support [6]. A tested tool is the wider distribution of positive examples and political empowerment of RD PAGs [23, 24].

### Strengths and shortcomings

Strengths of the study include the large number of participants reached through ePAGs’ channels, robust results, the comprehensive though brief nature of the survey, and the local input for translation by patients´ advocates. As a baseline collective action, the study played an important role in consolidating and mapping the roles of ePAGs representatives at Endo-ERN.

As stated above, the current survey does not represent the entire RD endocrine community in Europe, as specifically patients from Eastern European were underrepresented. Most likely, this reflects combinations of language barriers, different levels of maturity of professional patient organizations, if present at all, distrust in/lack of acceptance of EU policy, cultural differences, and other, as yet unidentified factors. The survey was not constructed to capture the degree of use of resources or the level of information of patients, or the differences between achievements of ePAGs in informing their membership/followers.

In conclusion, our current patient information access survey provides sound evidence for further planning and execution of educational and teaching activities by Endo-ERN.

## Supplementary information

Supplementary Table 1

Supplementary Information
