# Supplementary material for: Access to patient oriented information—a baseline Endo-ERN survey among patients with rare endocrine disorders
Source: Endocrine. 2021 Feb 18;71(3):542–8. doi: 10.1007/s12020-021-02654-9 (PMC8016814; doi:10.1007/s12020-021-02654-9)
Supplement: Supplementary file 1 — Supplementary Table 1 [file 12020_2021_2654_MOESM1_ESM.pdf]

1a. In which country do you live and have experience with a rare endocrine disease ?		
	Answers	Ratio
Netherlands	490	43,1%
France	234	20,6%
Germany	155	13,6%
Italy	58	5,1%
United Kingdom	50	4,4%
Finland	32	2,8%
Belgium	21	1,8%
Denmark	20	1,8%
Serbia	16	1,4%
Spain	15	1,3%
Austria	11	1,0%
Hungary	8	0,7%
Malta	8	0,7%
Bulgaria	4	0,4%
Sweden	4	0,4%
Greece	3	0,3%
Ireland	3	0,3%
Romania	2	0,2%
Luxembourg	1	0,1%
Portugal	1	0,1%
Slovenia	1	0,1%
Montenegro	1	0,1%
