# Supplementary material for: Access to patient oriented information—a baseline Endo-ERN survey among patients with rare endocrine disorders
Source: Endocrine. 2021 Feb 18;71(3):542–8. doi: 10.1007/s12020-021-02654-9 (PMC8016814; doi:10.1007/s12020-021-02654-9)
Supplement: Supplementary file 2 — Supplementary Information [file 12020_2021_2654_MOESM2_ESM.pdf]

ENDO-ERN - Survey about Patient informations in Europe

Fields marked with * are mandatory.

Please take a few moments to read the following before starting the survey:

What is the aim of this survey?

The aim of this survey is to gain the views and experiences of people living with rare endocrine disorders about the information about their disease within European countries in order to provide a better understanding patient needs and challenges.

Who should complete this questionnaire ?

This questionnaire is suitable only for adults aged 18 years and over. A parent or carer should complete this questionnaire on behalf of anyone under the age of 18. Please complete only one questionnaire per person. This survey should take no more than 5 minutes to complete.

Who is conducting this research?

This research is being conducted by the Education & Teaching working group of the European Reference network for rare endocrine conditions. (Endo-ERN)

What are European Reference Networks (ERN) ?

European Reference Networks (ERN) are virtual networks involving Reference Centers across Europe. They aim to tackle complex or rare medical diseases or conditions that require highly specialised treatment and a concentration of knowledge and resources.

What is Endo-ERN?

The European Reference Network on rare endocrine conditions (Endo-ERN) aims to improve access to high-quality healthcare for patients with hormonal disorders. Endocrine conditions are often complex and require a long period of care due to chronic disease without being life-threatening. Therefore, endocrine care requires equal distribution of paediatric and adult care. Endo-ERN aims to provide this care for patients throughout their entire lives and to reduce and ultimately abolish inequalities in care for patients with rare endocrine disorders in Europe, through facilitating knowledge sharing and facilitating related healthcare and research.The ENDO-ERN and and workgroups comply with the EU and national directives on data protection and personal privacy.

How will my answers be used?

The data collected in this research may be used by the ENDO-ERN and the Education&Teaching working group for a variety of activities. This may include, but is not limited to, articles for medical journals, patient group publications and campaigning or lobbying materials, presentations, social media campaigns, and so on.

Any information that you disclose will be treated in the strictest confidence. No answers will be attributable to you as an individual. You have the right to withdraw from the survey at any time.

By answering ‘yes’ to the question below, you confirm that you have read, understood and accept the points above.

* Are you happy to proceed on this basis?

- Yes
- No

ABOUT YOU

* 1a. In which country do you live and have experience with a rare endocrine disease ?

- Austria
- Belgium
- Bosnia and Herzegovina
- Bulgaria
- Croatia
- Cyprus
- Czech Republic
- Denmark
- Estonia
- Finland
- France
- Germany
- Greece
- Hungary
- Latvia
- Lithuania
- Luxembourg
- Macedonia
- Malta
- Montenegro
- Netherlands
- Poland
- Portugal
- Romania
- Serbia
- Slovak Republic
- Slovenia
- Spain

- Ireland
- Italy
- Sweden
- United Kingdom

* 1b. How old are you ? or Who old is your child ?

- 0 - 9
- 10 - 18
- 19 - 30
- 31 - 40
- 41 - 50
- 51 - 60
- 61 - 70
- over 70

* 1c. What gender do you identify with ?

- female
- male
- Prefer not to answer

* 2. Which type of rare endocrine disease are you referring to ?

To which area of rare endocrine diseases are the answers related?

- Adrenal
- Disorders of Calcium & Phospate Homeostasis
- Genetic Disorders of Glucose & Insulin Homeostasis
- Genetic Endocrine Tumour Syndromes
- Growth & Genetic Obesity Syndromes
- Pituitary
- Sex Development & Maturation
- Thyroid

Patient´s information

* 3. Where do you get the information about the rare disease?

- Specialist Reference Center
- General Practioner
- Endocrinologist
- Nurse
- Patient Advocacy Group
- Relative
- Social Media
- Website
- Other

If OTHER, please specify

* 3 a. Did you receive the information in your native language ?

- Yes
- No

4. How helpful are the following publication forms?

	not at all helpful	not so helpful	somewhat helpful	very helpful	extremely helpful
* Leaflet	☐	☐	☐	☐	☐
* Infographic	☐	☐	☐	☐	☐
* Internet Film	☐	☐	☐	☐	☐
* Webinar	☐	☐	☐	☐	☐

* 5. Which form do you use currently ?

- ☐ None, I don´t need the information
- ☐ None, there is no patient information about my disease
- ☐ Leaflet
- ☐ Infographic
- ☐ Internet Film
- ☐ Webinar
- ☐ Other

If OTHER, please specify:

* 6. Who published the information?

- ☐ Specialist in Reference Center
- ☐ Patient Advocacy Group
- ☐ Medical Journalist
- ☐ Pharmaceutical Company
- ☐ National Rare Disease Alliance
- ☐ Other

* 7. Which websites do you use currently?

- ☐ None
- ☐ National Patient Advocacy Group´s website
- ☐ Pharmaceutical Company´s website
- ☐ National Endocrine Society ´s website
- ☐ Foreign Patient Advocacy Group´s website
- ☐ EURORDIS
- ☐ Orphanet
- ☐ National Institutes of Health (NIH)
- ☐ Other

If OTHER, please specify:

* 8. Do you exchange general information about your disease via social media ?
- Very Unlikely
- Unlikely
- Neither
- Likely
- Very Likely

* 8a. Which kind of social media do you prefer?
- None
- Facebook
- Twitter
- Instragram
- Whats App group
- Other

If OTHER, please specifiy

* 9. Do you receive updates of your papers / informations ?
- Yes
- No
- There aren´t any updates

* 10. If you are a parent, would you like informational material, available for your children?
- Yes
- No
- I don´t know

* 11. Overall, how satisfied are you with the information about your rare endocrine disease?
- Very satisfied
- Somewhat satisfied
- Neither satisfied nor dissatisfied
- Somewhat dissatisfied
- Very dissatisfied

* 12. Would you like to engage in creating patient material?
- Yes
- No

* 12 a. What is your first choice if you are the creator?

If you would be interested in helping us with other similar research opportunities in the future, please leave us your email address below so that we can contact you.
Don't worry, we don't share your details with anyone else, nor will we send you loads of emails!

Email

You have reached the end of our survey - Thank you for participating.
